# On-Line Multi-Damage Scanning Spatial-Wavenumber Filter Based Imaging Method for Aircraft Composite Structure

**DOI:** 10.3390/ma10050519

**Published:** 2017-05-11

**Authors:** Yuanqiang Ren, Lei Qiu, Shenfang Yuan, Qiao Bao

**Affiliations:** Research Center of Structural Health Monitoring and Prognosis, State Key Lab of Mechanics and Control of Mechanical Structures, Nanjing University of Aeronautics and Astronautics, Nanjing 210016, China; renyuanqiang@nuaa.edu.cn (Y.R.); ysf@nuaa.edu.cn (S.Y.); baoqiao@nuaa.edu.cn (Q.B.)

**Keywords:** aircraft composite structure, structural health monitoring, damage imaging, spatial-wavenumber filter, guided wave

## Abstract

Structural health monitoring (SHM) of aircraft composite structure is helpful to increase reliability and reduce maintenance costs. Due to the great effectiveness in distinguishing particular guided wave modes and identifying the propagation direction, the spatial-wavenumber filter technique has emerged as an interesting SHM topic. In this paper, a new scanning spatial-wavenumber filter (SSWF) based imaging method for multiple damages is proposed to conduct on-line monitoring of aircraft composite structures. Firstly, an on-line multi-damage SSWF is established, including the fundamental principle of SSWF for multiple damages based on a linear piezoelectric (PZT) sensor array, and a corresponding wavenumber-time imaging mechanism by using the multi-damage scattering signal. Secondly, through combining the on-line multi-damage SSWF and a PZT 2D cross-shaped array, an image-mapping method is proposed to conduct wavenumber synthesis and convert the two wavenumber-time images obtained by the PZT 2D cross-shaped array to an angle-distance image, from which the multiple damages can be directly recognized and located. In the experimental validation, both simulated multi-damage and real multi-damage introduced by repeated impacts are performed on a composite plate structure. The maximum localization error is less than 2 cm, which shows good performance of the multi-damage imaging method. Compared with the existing spatial-wavenumber filter based damage evaluation methods, the proposed method requires no more than the multi-damage scattering signal and can be performed without depending on any wavenumber modeling or measuring. Besides, this method locates multiple damages by imaging instead of the geometric method, which helps to improve the signal-to-noise ratio. Thus, it can be easily applied to on-line multi-damage monitoring of aircraft composite structures.

## 1. Introduction

Structural health monitoring (SHM) has become a topic of great interest, since it is critical to increase the reliability and safety of structures and reduce maintenance costs, especially in aerospace engineering applications [1,2,3,4]. The increasing adoption of composite materials in various aircraft structures has presented a challenge to aerospace SHM. Falling tools, ground service carts, hailstones, loading abrasion and other objects may cause multiple damages during manufacturing, service and maintenance in the whole lifetimes of aircraft composite structures. These damages, such as matrix cracking, fiber breakage and inter-ply delamination and so on, may result in stiffness degradation and a serious loss of structural integrity [5,6,7]. Therefore, on-line multi-damage monitoring of composite structures has great significance in aircraft health monitoring.

Guided wave has proved to be particularly useful for SHM research due to their excellent propagation capability and high sensitivity to small defects [2,8,9]. In recent years, the guided wave based damage imaging method has emerged to be an interesting SHM topic. By combining with piezoelectric (referred as PZT) sensor array, this method is able to improve the signal-to-noise ratio when performing SHM applications for aircraft structures. According to the arrangement of the PZT array, these methods can be roughly divided into two kinds, in which the former kind of damage imaging methods are based on the sparse PZT array, such as delay-and-sum imaging [10,11,12], time reversal focusing imaging [13,14,15,16] and damage probability imaging [17,18,19,20]; the latter dense PZT array methods include ultrasonic phased array [21,22,23] and multi-signal classification [24,25,26] etc. These methods mainly concentrate on processing guided wave in time domain, frequency domain or time-frequency domain.

Compared with these methods, the spatial-wavenumber filter based method has proved to be an effective approach to distinguish propagating direction and different modes of the guided wave. Many researchers have tried to conduct multi-damage monitoring by taking advantages of the spatial-wavenumber filter technology [27,28,29,30,31]. For example, Sohn et al. [29] used a scanning laser Doppler vibrometer (SLDV) to obtain the wavefield image in spatial-wavenumber domain to isolate the standing wave components of a composite plate for delamination and disbond inspection. Bae et al. [30] developed a wavenumber filtering algorithm based multi-time-frame ultrasonic energy mapping method to measure multiple cracks in an in-service metallic aircraft fuselage structure. Kudela et al. [31] also adopted SLDV to obtain full wavefield data to estimate the length and orientation of multiple cracks in simple metallic and composite laminates.

However, all the research mentioned above adopted SLDV as the spatial sampling device, making them only applicable for off-line damage inspection. To conduct spatial-wavenumber filter based on-line damage monitoring, An et al. [32] proposed a crack diagnostic technique for an aluminum specimen, based on a special stripe-PZT sensor which includes two excitation PZTs and a linear PZT array used to measure spatial wavefield. Purekar et al. [33] carried out damage monitoring research of composite structures by replacing SLDV with a linear PZT array, but an accurate wavenumber curve is required to perform the spatial-wavenumber filtering. To avoid the wavenumber modeling or measuring, Qiu et al. [34] combined a PZT 2D cruciform array and a scanning spatial-wavenumber filter (SSWF) method to realize on-line damage imaging of composite structures. Although these methods have shown their potential, it can be seen that the current spatial-wavenumber filter based on-line damage monitoring method can only deal with the single damage situation.

Several problems still exist with the spatial-wavenumber filter when conducting on-line multi-damage monitoring of aircraft composite structures. Firstly, a multi-damage SSWF with wavenumber model-independent and measuring-independent needs to be established to directly perform spatial-wavenumber filtering of the multi-damage scattering signal. Secondly, the current on-line damage monitoring method based on spatial-wavenumber filter can only generate the wavenumber-time image. Accordingly, an additional geometric based method is needed to locate damage, resulting in a limited localization accuracy. In addition, the anisotropy of composite structures and the overlapping of wavenumbers of multi-damage scattering signal will further increase the difficulty in recognizing and locating the multiple damages.

In this paper, a new on-line multi-damage SSWF based imaging method is proposed to address the above-mentioned problems and realize on-line monitoring of multiple damages of aircraft composite structures. With this method, an on-line multi-damage SSWF based on a linear PZT array is established for the spatial-wavenumber filtering of the multi-damage scattering signal to get the wavenumber-time image without wavenumber modeling or measuring. Then an image-mapping method based on a PZT 2D cross-shaped array is proposed to perform wavenumber synthesis and convert the two wavenumber-time images obtained by the array to an angle-distance image so as to locate multiple damages. The multi-damage imaging method is validated on a composite plate structure and the imaging and localization of both simulated and real multiple damages are correctly achieved, respectively.

The rest of this paper is organized as follows. Section 2 presents the establishment of the on-line multi-damage SSWF based on a linear PZT array and the multi-damage wavenumber-time imaging method. In Section 3, a PZT 2D cross-shaped array based wavenumber synthesis mechanism is first introduced, followed by the angle-distance image-mapping method. A composite plate structure with both simulated and real damages is used to perform validation experiments to verify the proposed methods in Section 4. Finally, Section 5 gives discussion regarding the evaluation results.

## 2. On-Line Multi-Damage SSWF and Wavenumber-Time Imaging

In this section, a linear PZT array based SSWF is first established to perform spatial-wavenumber filtering of the multi-damage scattering signal. Then a multi-damage wavenumber-time imaging method without wavenumber modeling or measuring is proposed for the further localization processing of multiple damages.

### 2.1. On-Line Multi-Damage SSWF

A linear PZT array, which contains *M* PZTs with an equally small distance Δ*ρ*, is adopted to conduct spatial sampling. When performing on-line multi-damage monitoring, every PZT of the linear array acquires signals for a period of time. At each time point, the acquired signals of the *M* PZTs form a group of spatial signal, which has *M* data points and a sampling rate of 2π/Δ*ρ*.

**Figure 1:**
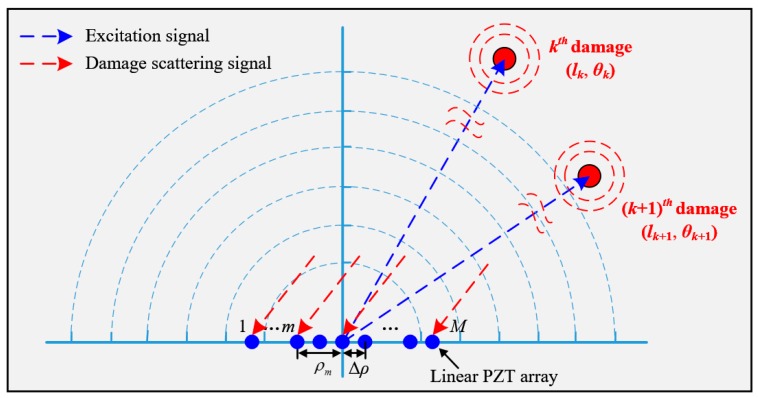
Schematic diagram of the spatial sampling based on a linear PZT array.

Figure 1 shows the schematic diagram of the multi-damage spatial sampling. A polar coordinate system is adopted to describe the positions of these damages. In this system, the pole coincides with the central point of the linear PZT array, the angle increases from 0° to 180° counterclockwise and the linear PZT array is along 0° direction.

**Figure 2:**
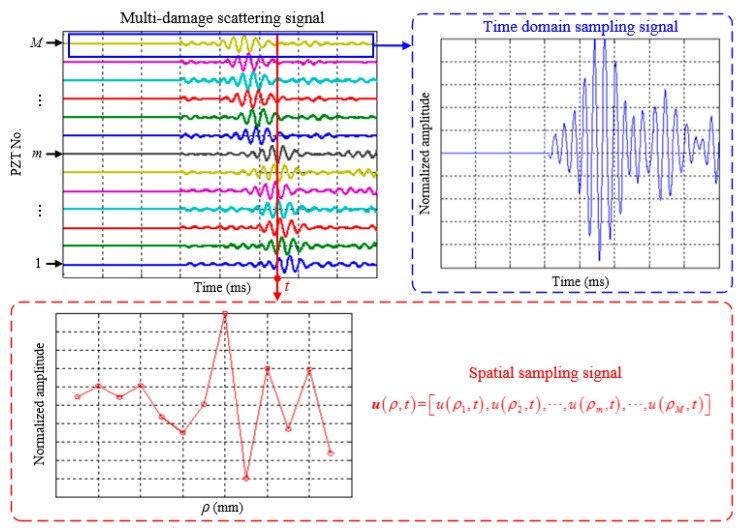
Demonstration of the spatial sampling signal.

Assuming there exist *K* damages in the structure, the distance and angle of the *k^th^* (1 ≤ *k* ≤ *K*) damage relative to the pole are denoted as *l_k_* and *θ_k_*, respectively, as shown in Figure 1. In order to locate these damages, the PZT located at the pole is used to excite a frequency narrow-band guided wave signal with a central frequency *ω_c_*. When the guided wave propagates to these damages, the multi-damage scattering signal, which consists of the guided waves induced by different damages is generated. The linear PZT array is used to acquire the multi-damage scattering signal. The spatial sampling signal of the multiple damages, namely the multi-damage scattering signal at time point *t*, can be expressed as Equation (1). To further explain the spatial sampling, Figure 2 gives out a typical example of the multi-damage scattering signal acquired by a linear PZT array, and compares the spatial signal with the time domain signal.

(1) $$u\left(\rho ,t\right)=\left[u\left(\rho_{1},t\right),u\left(\rho_{2},t\right),\cdots ,u\left(\rho_{m},t\right),\cdots ,u\left(\rho_{M},t\right)\right]$$

Because of the frequency narrow-band excitation signal, the multi-damage scattering signal is also considered to be a frequency narrow-band signal, and the wavenumbers of the scattering signals caused by different damages should be approximately equal. Therefore, the acquired signal of the No. *m* PZT can be expressed as Equation (2).

(2) $$u\left(\rho_{m},t\right)=\sum_{k=1}^{K}f_{m}^{k}(t)\cdot \exp(i\cdot (\omega_{c}t-\xi_{c}\left|{\overset{\text{→}}{l}}_{k}-{\overset{\text{→}}{\rho }}_{m}\right|))$$

where $f_{m}^{k}(t)$ is the amplitude of the scattering signal of the *k^th^* damage, *ξ_c_* is the wavenumber corresponding to *ω_c_*, ${\overset{\text{→}}{l}}_{k}$ and ${\overset{\text{→}}{\rho }}_{m}$ denote the distance vectors of *l_k_* and *ρ_m_*, respectively.

The phase change item $\left|{\overset{\text{→}}{l}}_{k}-{\overset{\text{→}}{\rho }}_{m}\right|$ in Equation (2) is replaced by its second-order Taylor expansion to simply Equation (2) into Equation (3).

(3) $$u\left(\rho_{m},t\right)=\sum_{k=1}^{K}f_{m}^{k}(t)\cdot \exp(i\cdot (\omega_{c}t-\xi_{c}l_{k}+\xi_{c}\cos\theta_{k}\rho_{m}-\frac{\xi_{c}\rho_{m}{\left(\sin\theta_{k}\right)}^{2}}{2l_{k}}))$$

In a far-field situation, the multi-damage scattering signal can be considered as a planar wave received by the linear PZT array. Hence Equation (3) can be further simplified [35].

(4) $$u\left(\rho_{m},t\right)=\sum_{k=1}^{K}f_{m}^{k}(t)\cdot \exp(i\cdot (\omega_{c}t-\xi_{c}l_{k}+\xi_{c}\cos\theta_{k}\rho_{m}))$$

Given the fact that the interval of every two PZTs is very small compared with distance of damage, the amplitude difference between the damage scattering signals acquired by different PZTs can be neglected. Therefore, the acquired signal of the No. *m* PZT is expressed as Equation (5).

(5) $$u\left(\rho_{m},t\right)=\sum_{k=1}^{K}f^{k}(t)\cdot \exp(i\cdot (\omega_{c}t-\xi_{c}l_{k}+\xi_{c}\cos\theta_{k}\rho_{m}))$$

To analyze the wavenumber characteristic of the spatial multi-damage scattering signal, the Discrete Fast Fourier Transform (DFFT) is adopted to transform the signal from spatial domain to wavenumber domain, as shown in Equations (6) and (7).

(6) $$\begin{array}{l} U\left(\xi ,t\right)=\sum_{\rho =\rho_{1}}^{\rho_{M}}\sum_{k=1}^{K}f^{k}(t)\cdot \exp(i\cdot (\omega_{c}t-\xi_{c}l_{k}+\xi_{c}\cos\theta_{k}\rho -\xi \rho ))\\ =\sum_{k=1}^{K}2\pi \cdot X^{k}(t)\cdot \delta (\xi -\xi_{c}\cos\theta_{k}) \end{array}$$

(7) $$X^{k}(t)=f^{k}(t)\cdot \exp(\omega_{c}-\xi_{c}l_{k})$$

where *δ* is the Dirac function given by Equation (8).

(8) $$\delta (\xi -\xi_{c}\cos\theta_{k})=\left\{ \begin{array}{l} \begin{array}{cc} 1 & \xi =\xi_{c}\cos\theta_{k} \end{array}\\ \begin{array}{cc} 0 & \xi \neq \xi_{c}\cos\theta_{k} \end{array} \end{array}\right.$$

**Figure 3:**
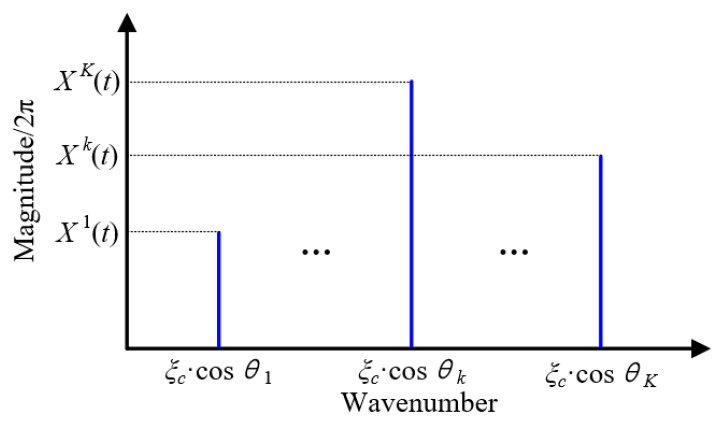
Wavenumber spectrum of the spatial signals acquired by the linear PZT array.

Figure 3 shows the wavenumber spectrum of the spatial signals obtained based on Equation (6). It can be seen from the figure that there are *K* discrete wavenumbers corresponding to the *K* damages. These wavenumbers are projection wavenumbers on the linear PZT array of different damages with different angles. For example, the projection wavenumber of the *k^th^* damage with angle *θ_k_* is *ξ_c_·*cos*θ_k_*. After obtaining the wavenumber spectrum, a spatial-wavenumber filter is designed by combining with the linear PZT array, given by Equations (9) and (10).

(9) $$\phi \left(\rho ,\xi_{n}\right)=\left[\phi \left(\rho_{1},\xi_{n}\right),\phi \left(\rho_{2},\xi_{n}\right),\cdots ,\phi \left(\rho_{m},\xi_{n}\right),\cdots ,\phi \left(\rho_{M},\xi_{n}\right)\right]$$

(10) $$\phi \left(\rho_{m},\xi_{n}\right)=\exp(i\xi_{n}\rho_{m})$$

where *ξ_n_* represents the central wavenumber of this filter. As shown in Equation (11), its wavenumber spectrum is also obtained by DFFT, which indicates that the filter is able to selectively allow the signal with the wavenumber of *ξ_n_* to pass, while reject the signals with other wavenumbers.

(11) $$\Phi \left(\xi \right)=2\pi \delta \left(\xi -\xi_{n}\right)$$

Since the wavenumber *ξ*_c_ is unknown without modeling or measuring in actual situation, a series of the above-mentioned spatial-wavenumber filters are designed to construct a matrix, which is called as SSWF and is given in Equation (12). Every row of the matrix represents a spatial-wavenumber filter with its own central wavenumber. In addition, the wavenumber of each row is in ascending order, from the minimum wavenumber to the maximum wavenumber that the linear PZT array can handle.

(12) $$\phi \left(\rho ,\xi_{n}\right)={\left[\begin{array}{cccc} \phi \left(\rho_{1},\xi_{1}\right) & \phi \left(\rho_{2},\xi_{1}\right) & \cdots & \phi \left(\rho_{M},\xi_{1}\right)\\ \vdots & \vdots & \ddots & \vdots \\ \phi \left(\rho_{1},\xi_{n}\right) & \phi \left(\rho_{2},\xi_{n}\right) & \cdots & \phi \left(\rho_{M},\xi_{n}\right)\\ \vdots & \vdots & \ddots & \vdots \\ \phi \left(\rho_{1},\xi_{N}\right) & \phi \left(\rho_{2},\xi_{N}\right) & \cdots & \phi \left(\rho_{M},\xi_{N}\right) \end{array}\right]}_{N\times M},n=1,2\cdots ,N$$

### 2.2. Multi-Damage Wavenumber-Time Imaging Method

**Figure 4:**
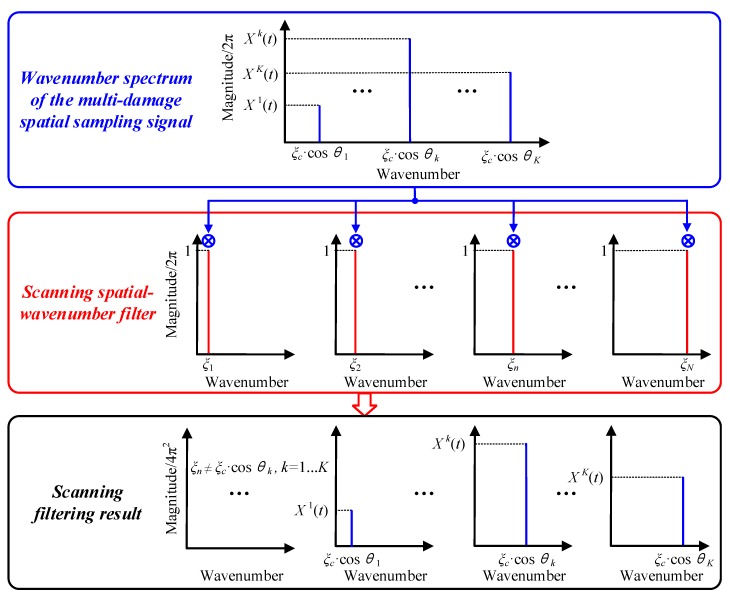
Schematic of the SSWF for multi-damage.

After applying the established SSWF to the multi-damage spatial sampling signal, the scanning filtering response is obtained and expressed as Equation (13). Figure 4 gives out the implementation process of the SSWF. According to Equation (13), if the scanning wavenumber is equal to the projection wavenumber of the *k^th^* damage on the linear PZT array, namely *ξ_n_ = ξ_c_*·cos*θ_k_*, the spatial sampling signal corresponding to the *k^th^* damage is able to pass the SSWF, generating a large ***H***(*t, ξ_n_*) value. By contrast, the spatial sampling signal will be rejected by the SSWF when *ξ_n_ ≠ ξ_c_*·cos*θ_k_*, generating a small ***H***(*t, ξ_n_*) value. In general, the SSWF is able to recognize the projection wavenumbers of the multi-damage spatial sampling signal on the linear PZT array.

(13) $$H\left(t,\xi_{n}\right)=u\left(\rho ,t\right)⊗\phi {\left(\rho ,\xi_{n}\right)}^{T}=\left|\sum_{k=1}^{K}4\pi^{2}\cdot X^{k}\cdot \delta (\xi_{n}-\xi_{c}\cdot \cos\theta_{k})\right|$$

The maximum wavenumber *ξ*_max_ of the SSWF mentioned above is decided by the spatial sampling rate of the linear PZT array. Similar with the sampling theorem of time domain, *ξ*_max_ should be less than half of the spatial sampling rate. Equation (15) gives the maximum scanning step *N*. Δ*ξ* is the scanning resolution. Then the scanning wavenumber *ξ_n_*, namely the central wavenumber of the SSWF is expressed as Equation (16). The scanning scope of the filter is from −*ξ*_max_ to *ξ*_max_.

(14) $$\xi_{\max}<\frac{1}{2}\cdot \frac{2\pi }{\Delta \rho }=\frac{\pi }{\Delta \rho }$$

(15) $$N=\frac{2\xi_{\max}}{\Delta \xi }+1$$

(16) $$\xi_{n}=-\xi_{\max}+\left(n-1\right)\Delta \xi ,\;n=1,2,\cdots ,N$$

**Figure 5:**
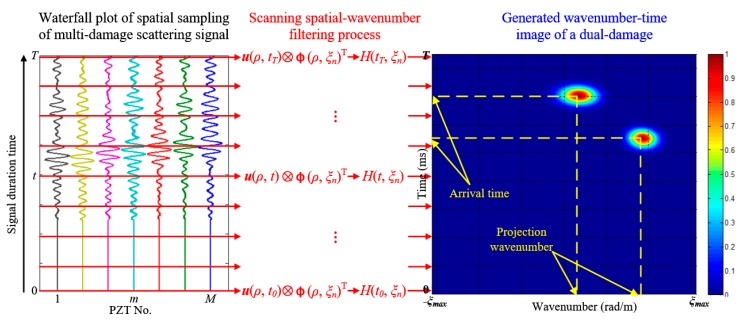
The wavenumber-time imaging process of a sample dual-damage.

Assuming the multi-damage scattering signal has a time length of *T*, then a response matrix **H** can be obtained after scanning filtering all the spatial sampling signals from time point 0 to *T*. By normalizing every element in **H** and imaging, a wavenumber-time image is generated. Figure 5 gives out the wavenumber-time imaging process of a dual-damage as an example. The generated wavenumber-time image is a kind of speckle image. The range of abscissa wavenumber is from −*ξ*_max_ to *ξ*_max_ and the ordinate time is from 0 to *T*. In this image, every pixel point represents the magnitude of the filtering output at specific scanning wavenumber and time point. There are two speckles in the wavenumber-time image, corresponding to the two damages. Each speckle contains the information of the damage’s projection wavenumber and arrival time.

## 3. The SSWF Based Multi-Damage Mapping Imaging

Based on the above research, the wavenumber-time image with the projection wavenumbers and arrival time of multiple damages can be obtained. According to this, a SSWF based multi-damage imaging method is proposed to recognize and locate the multiple damages, which mainly consists of the following two parts.

(1) A PZT 2D cross-shaped array is adopted to fulfill the scanning spatial-wavenumber filtering of the multi-damage scattering signal and wavenumber synthesis, which is necessary to realize the multi-damage mapping imaging.
(2) Based on the generated wavenumber-time image and wavenumber, an imaging mapping method is proposed to convert the wavenumber-time image to an angle-distance image, from which the multiple damages can be directly located.

### 3.1. PZT 2D Cross-Shaped Array Based Wavenumber Synthesis

**Figure 6:**
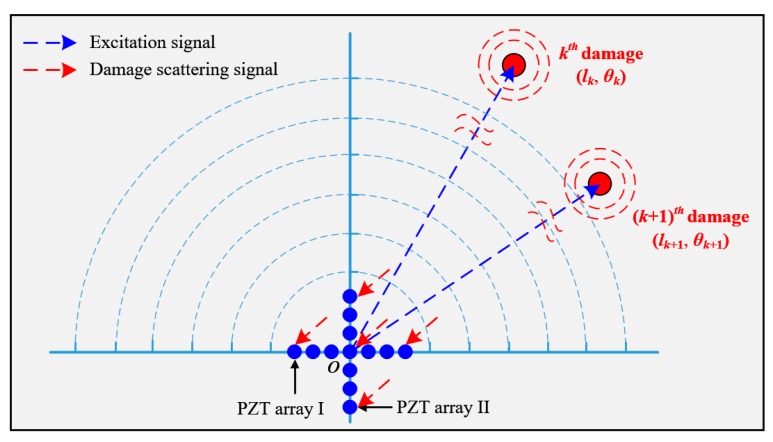
Schematic diagram of spatial sampling with a PZT 2D cross-shaped array.

As shown in Figure 6, the PZT 2D cross-shaped array contains two orthogonal linear PZT arrays labeled as No. I and No. II. A polar coordinate system is built and the central point of the 2D cross-shaped array is set to be the pole. PZT array I and II have the same PZT distribution and share the same central PZT, but the former is along 0° direction and the latter is along 90° direction.

The frequency narrow-band guided wave signal is excited at the pole, and the PZT 2D cross-shaped array is used to fulfill spatial sampling of the multi-damage scattering signal. According to Equation (12), a multi-damage SSWF can be established. After conducting the SSWF on the PZT 2D cross-shaped array, two wavenumber-time images of PZT array I and II can be obtained. The projection wavenumbers *ξ_k_^I^* and *ξ_k_^II^* of the *k^th^* damage on the two linear PZT arrays can be expressed as Equation (17), meaning that *ξ_k_^I^* and *ξ_k_^II^* are the cosine value and sine value of the wavenumber *ξ_c_*, respectively.

(17) $$\left\{ \begin{array}{l} \xi_{k}^{I}=\xi_{c}\cdot \cos\theta_{k}\\ \xi_{k}^{II}=\xi_{c}\cdot \cos\left(90^{\circ }-\theta_{k}\right)=\xi_{c}\cdot \sin\theta_{k} \end{array}\right.$$

**Figure 7:**
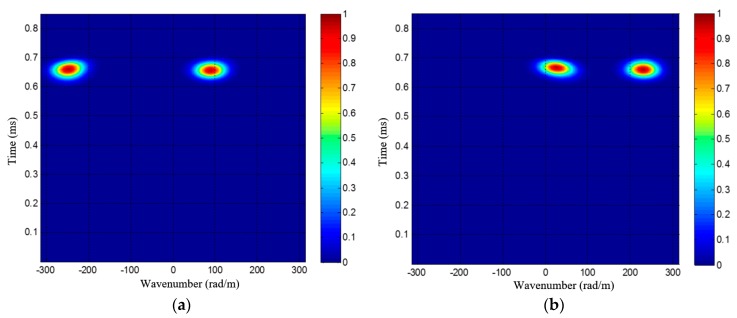
PZT 2D cross-shaped based wavenumber-time imaging result of a sample dual-damage; (**a**) Wavenumber-time image of PZT array I; (**b**) Wavenumber-time image of PZT array II.

Figure 7 gives out two wavenumber-time images of a dual-damage as an example, each wavenumber-time image has two speckles, corresponding to the two damages. It is unable to directly recognize and locate these damages by the two wavenumber-time images, unlike the single damage situation [34]. As shown in Figure 7, the speckles of different damages may have the same arrival time, making it hard to find the one-to-one relationship of every damage’s two speckles in the two wavenumber-time images. In order to address this issue, a wavenumber synthesis method is proposed first. Then the generated wavenumber is used in an angle-distance image-mapping method for the multi-damage localization, which will be discussed in detail in Section 3.2.

Considering that the multi-damage scattering signal is a frequency narrow-band signal, the wavenumbers of different damages should be equal to each other. Therefore, based on Equation (17), the wavenumber can be expressed as follows.

(18) $$\xi_{c}=\sqrt{{\left(\xi_{1}^{I}\right)}^{2}+{\left(\xi_{1}^{II}\right)}^{2}}=\cdots =\sqrt{{\left(\xi_{k}^{I}\right)}^{2}+{\left(\xi_{k}^{II}\right)}^{2}}=\cdots =\sqrt{{\left(\xi_{K}^{I}\right)}^{2}+{\left(\xi_{K}^{II}\right)}^{2}}$$

where *ξ_k_^I^* and *ξ_k_^II^* represent the projection wavenumbers of the *k^th^* damage on PZT array I and II, respectively.

Due to the one-to-one relationship issue mentioned before, it is hard to directly calculate the wavenumber *ξ_c_* by Equation (18) when there exist multiple damages. In order to solve this problem, Equation (19) is developed to calculate *ξ_c_*, which is able to avoid the one-to-one correspondence issue and obtain a relatively accuracy wavenumber.

(19) $$\xi_{c}=\frac{1}{K}\cdot \sum_{k=1}^{K}\sqrt{{\left(\xi_{k}^{I}\right)}^{2}+{\left(\xi_{k}^{II}\right)}^{2}}=\sqrt{\frac{1}{K}\cdot \left[\sum_{k=1}^{K}{\left(\xi_{k}^{I}\right)}^{2}+\sum_{k=1}^{K}{\left(\xi_{k}^{II}\right)}^{2}\right]}$$

It can be seen that the number of damages *K* is necessary to the calculation of *ξ_c_*. Normally, there will exist multiple speckles in the wavenumber-time image, corresponding to the multiple damages, as shown in Figure 7. *K* can be simply estimated by counting the number of speckles in the wavenumber-time image. Then *ξ_c_* is also obtained, according to Equation (19).

### 3.2. Angle-Distance Image Mapping Method

Based on the wavenumber *ξ_c_*, the angle-distance image-mapping method can be conducted. The basic principle of the method is calculating the unknown pixel values of all the pixel points in the angle-distance image, according to the two known wavenumber-time images. During this process, every pixel point in the angle-distance image is considered to be a possible damage source with its own distance and arrival time. For a point (*l*, *θ*) in the angle-distance image, it has two projection points (*t^I^*, *ξ^I^*) and (*t^II^*, *ξ^II^*) in the wavenumber-time images of PZT array I and II, respectively. Based on Equation (17), the mapping relationship between *θ* and *ξ^I^* or *ξ^II^* is achieved. As to the relationship between *l* and *t^I^* or *t^II^*, it should be noted that PZT array I and II share the same central PZT, as shown in Figure 6, the arrival time *t^I^* and *t^II^* should be equal, labeled as *t*. Besides, each arrival time *t* in the wavenumber-time image contains two parts. The first part is the guided wave excitation time *t_e_* decided by the excitation signal. The second part is the actual time-of-flight *t_a_*, including the time of guided wave signal propagating from the excitation position to the damage position and the time of the damage scattering signal propagating from the damage position to the 2D cross-shaped array. With the guided wave group velocity *v_g_*, the mapping relationship between *l* and *t* can be calculated by Equation (20).

(20) $$t=t_{e}+t_{a}=t_{e}+\frac{2l}{v_{g}}$$

Then, the two projection points of (*l*, *θ*) in the wavenumber-time images of PZT array I and II are (*t_e_ +* 2*l/v_g_*, *ξ_c_*·cos*θ*) and (*t_e_ +* 2*l/v_g_*, *ξ_c_*·sin*θ*), respectively. In addition, its pixel value **E**(*l*, *θ*) can be obtained by summing the pixel values of the two projection points, defined as Equation (21). The larger its pixel value is, the more likely it can be considered as an actual damage source.

(21) $$E\left(l,\theta \right)=H^{I}(t_{e}+2l/v_{g},\xi_{c}\cdot \cos\theta )+H^{\mathrm{II}}(t_{e}+2l/v_{g},\xi_{c}\cdot \sin\theta )$$

where **E** represents the angle-distance image, **H^I^** and **H^II^** represent the wavenumber-time images of PZT array I and II, respectively.

**Figure 8:**
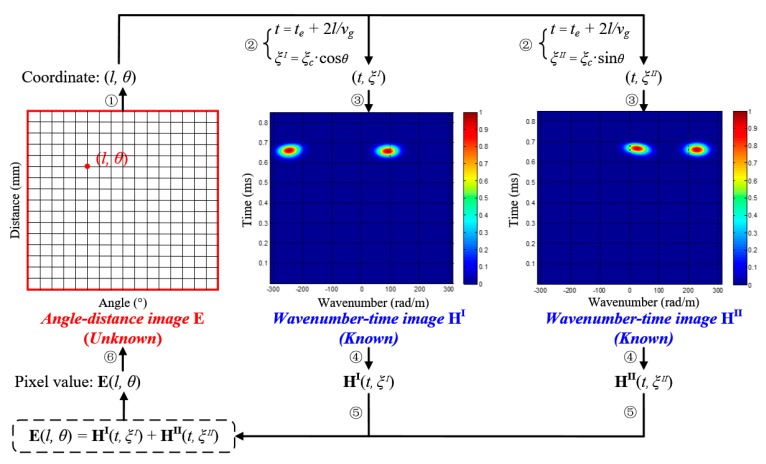
Conversion from the wavenumber-time image to angle-distance image.

The schematic diagram of the conversion from wavenumber-time image to angle-distance image is given out in Figure 8. Repeating this process and calculating the pixel values of all the points, the angle-distance image is achieved. Then the multiple damages can be recognized manually and their polar coordinates can be directly obtained from the angle-distance image.

### 3.3. Implementation Process of the Multi-Damage Imaging Method

**Figure 9:**
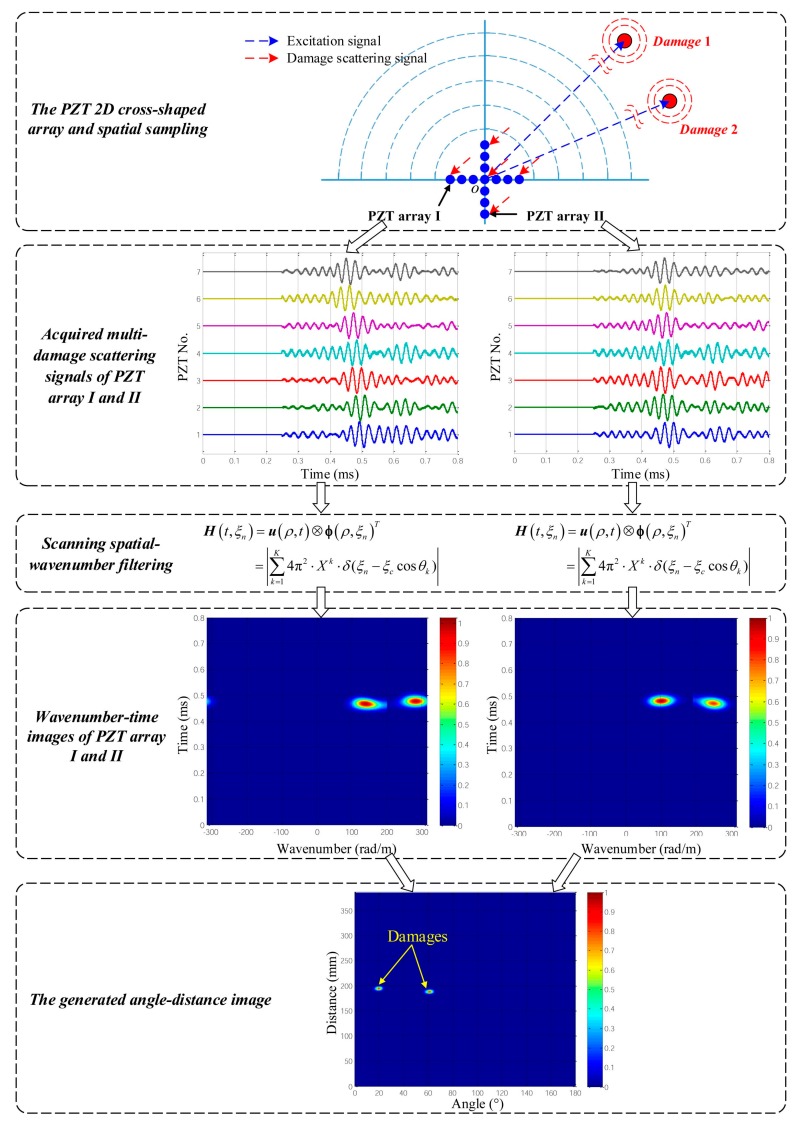
Implementation process of the proposed multi-damage imaging method.

According to the research mentioned above, the proposed SSWF based multi-damage imaging method is realized by using the PZT 2D cross-shaped array. Taken a dual-damage as an example, Figure 9 gives out the specific implementation process, which mainly consists of the following parts.

(1) Acquire the multi-damage scattering signals by adopting a PZT 2D cross-shaped array.
(2) Establish a multi-damage SSWF and then apply it to the acquired multi-damage scattering signals, according to Equation (13).
(3) Based on the scanning spatial-wavenumber filtering result, generate two wavenumber-time images and calculate the wavenumber *ξ_c_* of the multi-damage scattering signals, according to Equation (19).
(4) By taking advantage of *ξ_c_* and the two wavenumber-time images, convert the wavenumber-time images to an angle-distance image based on Equation (21), and then directly locate the multiple damages.

## 4. Experimental Validations

### 4.1. Validation Setup

**Figure 10:**
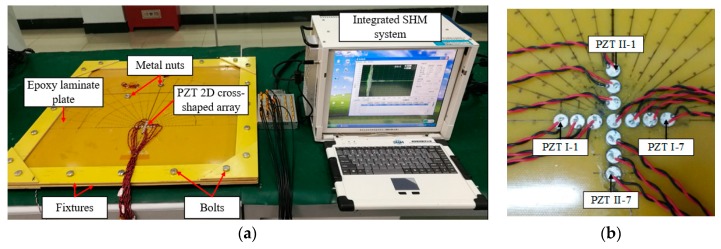
The validation system. (**a**) Experimental devices; (**b**) PZT 2D cross-shaped array.

Figure 10a shows the experimental system, which mainly contains an epoxy laminate plate, a PZT 2D cross-shaped array and an integrated SHM system (SHM-ISS-4.0C, NUAA, Nanjing, China) [36]. The plate has a dimension of 600 mm × 600 mm × 3 mm (Length × Width × Thickness) and its ply sequence is [0_2_/90_4_/0_2_]_2S_. The 4 sides of the plate are fixed between 2 fixtures by a series of bolts. Supported by the bolts, the plate and the fixtures are placed on a table horizontally, and do not directly contact the table.

As shown in Figure 10b, a PZT 2D cross-shaped array is placed on the structure and is used to fulfill spatial sampling. This 2D array consists of 2 orthogonal linear PZT arrays, and each linear array has 7 PZT sensors with a diameter of 8 mm and a thickness of 0.48 mm. The interval between every two adjacent PZT sensors is Δ*ρ* = 10 mm. The PZTs in PZT array I are labeled as PZT I-1, PZT I-2, …, PZT I-7 and those in PZT array II are labeled as PZT II-1, PZT II-2, …, PZT II-7. PZT I-4 and PZT II-4 are the same one. Besides, an actuator that is used to excite guided wave is placed at the central point of the PZT 2D cross-shaped array on the back side of the structure.

The integrated SHM system is used to control the actuator to generate guided wave in the structure and the PZT 2D cross-shaped array to acquire signals. The excitation signal is a five-cycle sine burst modulated by the Hanning window with amplitude of ±70 V. The signal’s central frequency is set to be 35 kHz, so that the corresponding wavenumber of the signal is less than the maximum scanning wavenumber *ξ*_max_, and the time domain resolution of the wavenumber-time image is not too low to identify the arrival time and distinguish different damages. The sampling rate is set to be 10 MS/s, and the sampling length is 8000 samples, including 1500 pre-trigger samples. The trigger voltage is 6 V.

The experimental validations in this work adopt both simulated damages and real damages to verify the proposed multi-damage imaging method. First, the metal nut with a diameter of 14 mm is used to change the local stiffness of the structure to simulate damage. For example, as shown in Figure 10a, two metal nuts fixed at different positions by adhesive are used to simulate a dual-damage situation. Second, after the simulated damages tests, two real damages are introduced to the structure by repeated impacts at particular positions.

### 4.2. Validation Results of Simulated Damages

**Figure 11:**
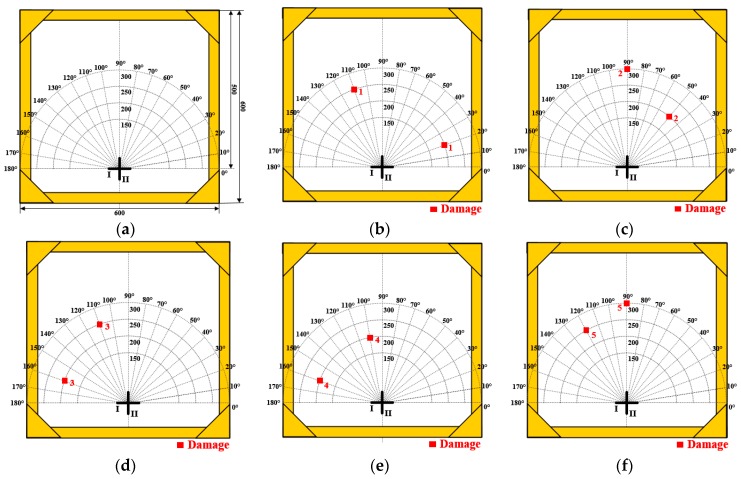
Schematic diagram of the structure and the 5 dual-damage situations. (**a**) Schematic of the structure; (**b**) The 1st dual-damage; (**c**) The 2nd dual-damage; (**d**) The 3rd dual-damage; (**e**) The 4th dual-damage; (**f**) The 5th dual-damage.

In this section, a total of 5 simulated dual-damage situations and a trinal-damage situation are tested. Taking the center of PZT I-4/II-4 as the pole, a polar coordinate system is built. Table 1 lists the polar coordinates of the 5 dual-damage situations. In Figure 11a, the schematic diagram of the structure is given out, Figure 11b,f show the distribution of the 5 dual-damage situations.

**Figure 12:**
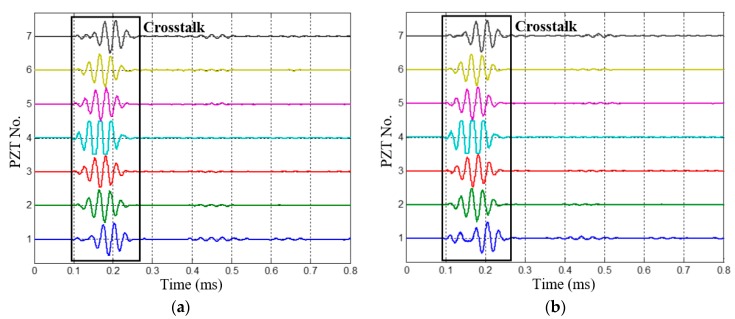
The health reference signals of the 1st dual-damage situation. (**a**) Signals of PZT array I; (**b**) Signals of PZT array II.

**Figure 13:**
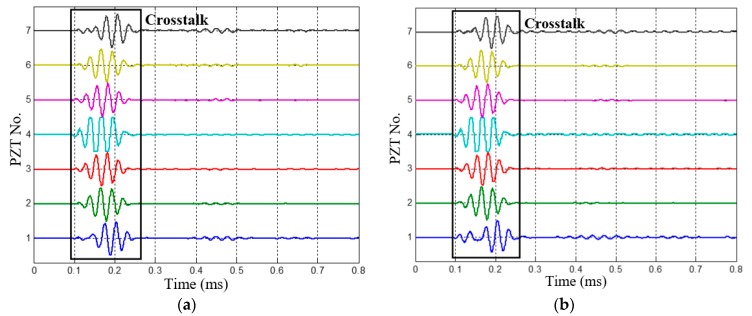
The on-line monitoring signals of the 1st dual-damage situation. (**a**) Signals of PZT array I; (**b**) Signals of PZT array II.

**Figure 14:**
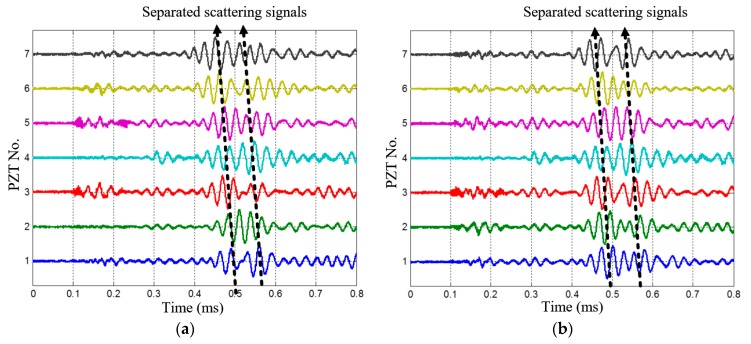
Multi-damage scattering signals of the 1st dual-damage situation. (**a**) Multi-damage scattering signals of PZT array I; (**b**) Multi-damage scattering signals of PZT array II.

The 1st dual-damage, which has two damages located at (200 mm, 20°) and (250 mm, 110°), is chosen as a typical case to be analyzed. The health reference signals and on-line monitoring signals of the 1st dual-damage acquired by the PZT 2D cross-shaped array are shown in Figure 12 and Figure 13, respectively. By subtracting them, the multi-damage scattering signals are obtained and shown in Figure 14. It can be seen that the scattering signals induced by the two damages are obviously separated. Considering the low central frequency of 35 kHz, the signals should be A0 mode while the S0 mode is barely visible. What should be also noted is that the signals around 0.2 ms are just the difference signals of crosstalk between the health reference signals and on-line monitoring signals.

**Figure 15:**
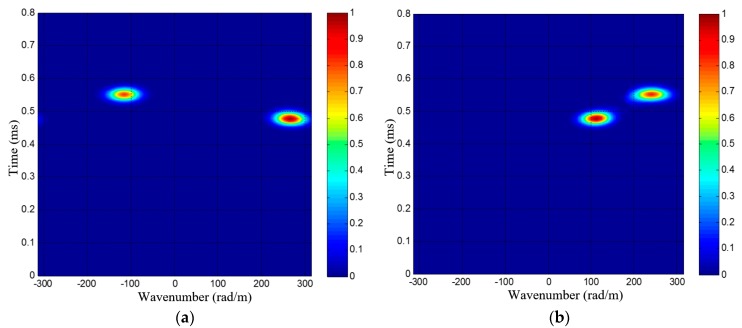
The wavenumber-time images of the 1st dual-damage situation. (**a**) Wavenumber-time image of PZT array I; (**b**) Wavenumber-time image of PZT array II.

Since Δ*ρ* = 10 mm, the maximum scanning wavenumber of the SSWF is *ξ*_max_ = 314 rad/m, according to Equation (14). After applying the SSWF to the multi-damage scattering signals, the wavenumber-time images of PZT array I and II are both obtained, as shown in Figure 15.

According to Figure 15, the wavenumber *ξ_c_* of the 1st dual-damage situation is available. First, two speckles can be recognized in each of the wavenumber-time images, so the number of damages is considered to be *K* = 2. Second, the projection wavenumbers and arrival time of the two damages are (−116 rad/m, 0.5435 ms) and (265 rad/m, 0.4784 ms) in the wavenumber-time image of PZT array I, and they are (111 rad/m, 0.4777 ms) and (240 rad/m, 0.5451 ms) in the wavenumber-time image of PZT array II. Based on the estimated number of damages and Equation (19), the wavenumber of the multi-damage scattering signals is calculated as 277 rad/m.

In order to convert the wavenumber-time images mentioned above to an angle-distance image to locate the multiple damages, the guided wave excitation time *t_e_* is needed. By using the continuous complex Shannon wavelet transform, the envelope of the signal can be obtained. The time corresponding to the maximum value of the envelope is considered to be the excitation time, which is *t_e_* = 0.1551 ms. Besides, the group velocity *v_g_* of the guided wave propagating in the structure is also necessary to conduct the image conversion. In this paper, *v_g_* is obtained through measuring and averaging the group velocities of the modulated five-cycle sine burst signals excited at different directions. When the central frequency is 35 kHz, *v_g_* = 1198.9 m/s.

**Figure 16:**
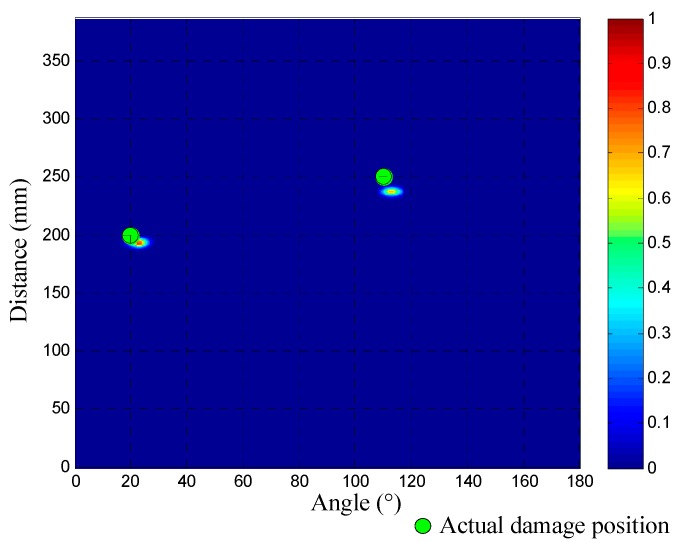
Angle-distance imaging and localization result of the 1st dual-damage situation.

Then, the two wavenumber-time images of PZT array I and II can be converted to an angle-distance image based on the mapping method proposed in Section 3.2. Figure 16 gives out the generated angle-distance image, in which two damages can be directly recognized and located. The two damages’ polar coordinates are (193.8 mm, 22.7.0°) and (238.8 mm, 112.1°). The green circles in Figure 16 represent the actual positions of the two damages for reference. The localization errors of the two damages are 11.2 mm and 14.3 mm, respectively.

**Figure 17:**
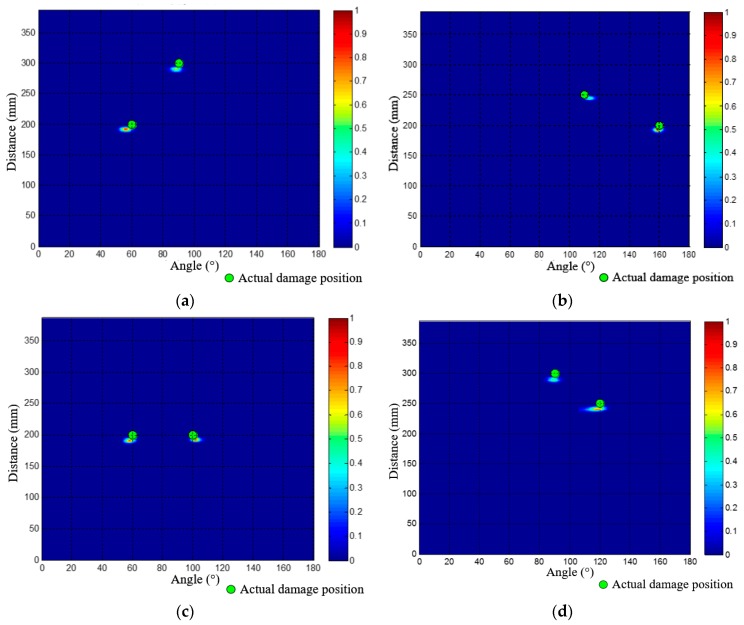
Angle-distance images of the other four dual-damage situations. (**a**) Angle-distance image of the 2nd dual-damage; (**b**) Angle-distance image of the 3rd dual-damage; (**c**) Angle-distance image of the 4th dual-damage; (**d**) Angle-distance image of the 5th dual-damage.

Based on the locating process mentioned above, the angle-distance images of the other 4 dual-damage situations are also obtained, as shown in Figure 17. The localization results and errors of all the 5 dual-damage situations are listed in Table 2, all the dual-damage situations are in good agreement with their actual positions.

**Figure 18:**
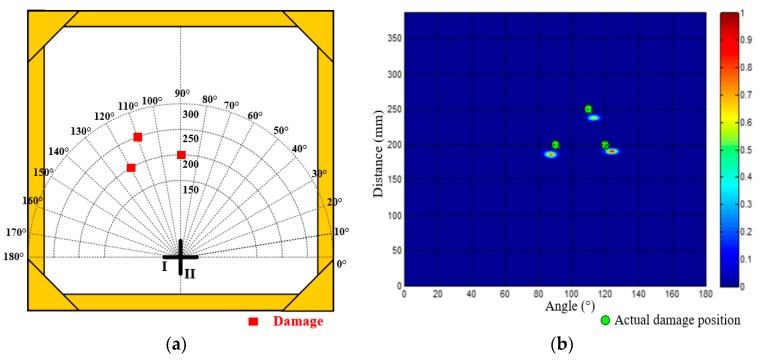
Distribution of the trinal-damage and its imaging result. (**a**) Positions of the three damages; (**b**) Angle-distance image of the trinal-damage.

Besides the 5 dual-damage situations, a trinal-damage test is also performed here. As shown in Figure 18a, the polar coordinates of the three damages are (200 mm, 90°), (200 mm, 120°) and (250 mm, 110°). Figure 18b gives out the imaging and localization result. The localization errors of the three damages are 15.8 mm, 16.5 mm and 18.6 mm, showing that the prosed method also works for the trinal-damage situation. According to all the verification results, it can be seen that the maximum localization error is less than 2 cm.

### 4.3. Validation Results of Real Damages

**Figure 19:**
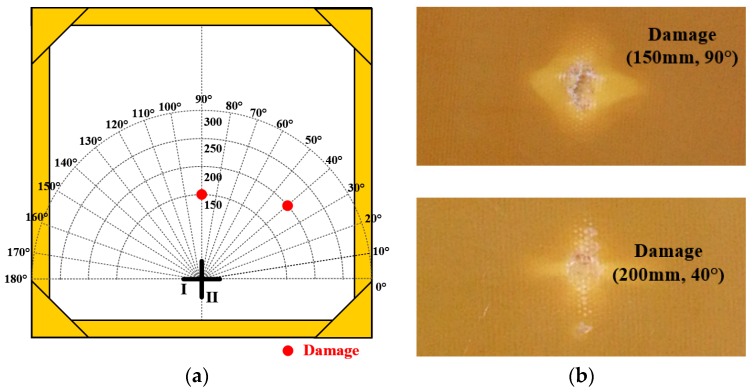
Distribution and exhibition of the two real damages. (**a**) Damages distribution on the structure; (**b**) Exhibition of the two damages caused by impact.

In order to further verify the effectiveness of the presented methods, two real impact damages located at (150 mm, 90°) and (200 mm, 40°) are introduced to the structure by repeated impacts with a hammer, as shown in Figure 19. The diameter of the two impact damages is about 10 mm.

**Figure 20:**
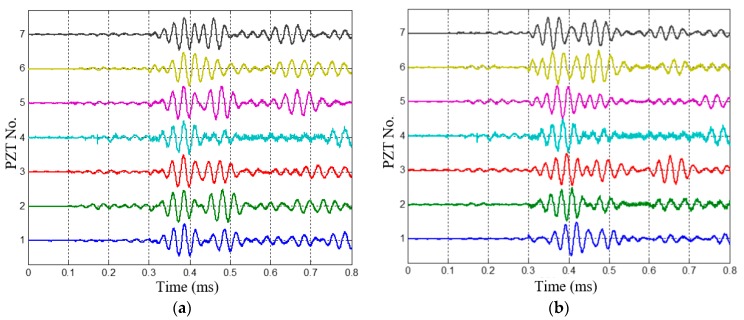
Acquired multi-damage scattering signals. (**a**) Multi-damage scattering signals of PZT array I; (**b**) Multi-damage scattering signals of PZT array II.

**Figure 21:**
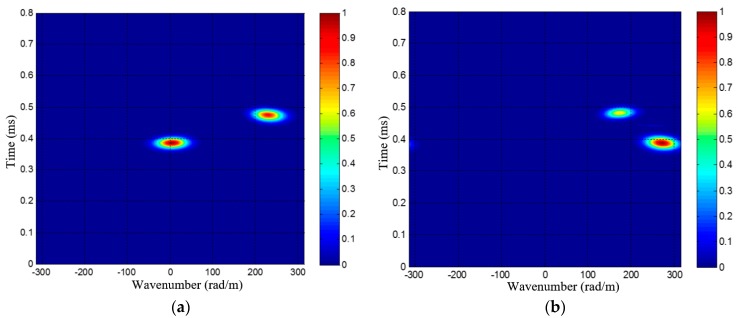
Wavenumber-time images of the two real impact damages. (**a**) Wavenumber-time image of PZT array I; (**b**) Wavenumber-time image of PZT array II.

**Figure 22:**
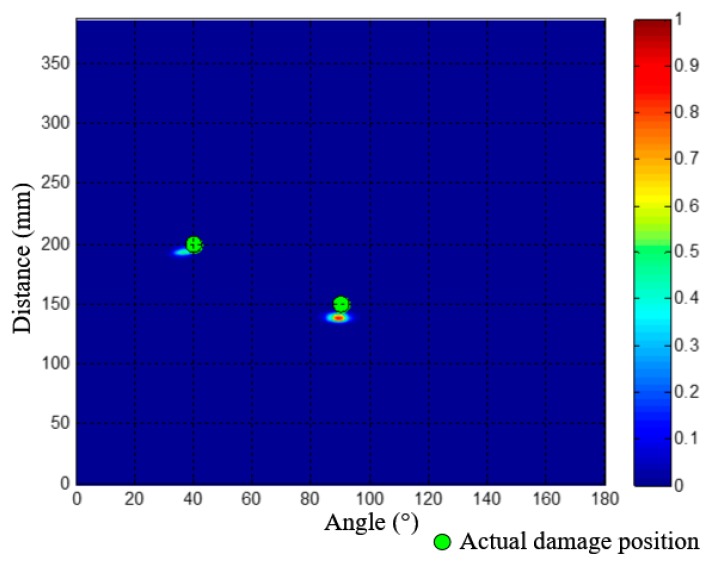
Angle-distance image of the two real impact damages.

As shown in Figure 20, the multi-damage scattering signals of the two real damages can be obtained by the PZT 2D cross-shaped array. Figure 21 gives out the two wavenumber-time images of PZT array I and II generated by applying the designed SSWF to the multi-damage scattering signals. In addition, the wavenumber *ξ*_c_ is calculated as 278 rad/m. Based on the proposed imaging mapping method, the wavenumber-time images are converted to an angle-distance image, as shown in Figure 22. According to this image, the two real damages are located at (138.5 mm, 89.4°) and (193.1 mm, 36.6°), and the localization errors of them are 11.6 mm and 13.5 mm, respectively, which proves the effectiveness of the multi-damage SSWF based imaging method proposed in this paper.

## 5. Conclusions

This paper proposes an on-line multi-damage scanning spatial-wavenumber filter based imaging method for aircraft composite structures, by taking advantages of the spatial-wavenumber filter technique. This method establishes an on-line multi-damage SSWF to realize the implementation of scanning spatial-wavenumber filtering and the generation of wavenumber-time image. By combining the multi-damage SSWF and a PZT 2D cross-shaped array, two wavenumber-time images and the wavenumber of the multi-damage scattering signal can be obtained, based on which an angle-distance mapping imaging method is presented. From the generated angle-distance image, the multiple damages can be directly recognized and located. Five simulated dual-damage situations, a trinal-damage situation and a real dual-damage introduced by repeated impacts are performed on a composite plate structure to conduct experimental validations. The maximum damage localization error is less than 2 cm, showing the effectiveness of the proposed on-line multi-damage scanning spatial-wavenumber filter based imaging method. In addition, without depending on any wavenumber modeling or measuring, the method has proved to be applicable to on-line multi-damage monitoring of aircraft composite structures.

However, the theoretical fundamental of the proposed multi-damage imaging method is considered in the far-field situation, which limits the performance of this method since damages may also exist in near-field. In addition, during the image-mapping process, the estimation of distance relies on the group velocity of guided wave, which is an average velocity in this paper and may cause localization error. Thus, ongoing work will concentrate on the development of a full-field SSWF and eliminate the dependence on wave velocity, and further verify the method on more complex aircraft composite structures.

## Figures and Tables

**Table 1:** Polar coordinates of the 5 simulated dual-damage situations.

Dual-Damage No.	Dual-Damage Position (mm, °)	
	Damage 1	Damage 2
1	(200, 20)	(250, 110)
2	(200, 60)	(300, 90)
3	(250, 110)	(200, 160)
4	(200, 100)	(200, 60)
5	(250, 120)	(300, 90)

**Table 2:** Statistical localization results of the 5 dual-damage situations.

No.	Damage 1			Damage 2		
	Actual Position (mm, °)	Localization Result (mm, °)	Position Error (mm)	Actual Position (mm, °)	Localization Result (mm, °)	Position Error (mm)
1	(200, 20)	(193.8, 22.7)	11.2	(250, 110)	(238.8, 112.1)	14.3
2	(200, 60)	(190, 56.5)	15.6	(300, 90)	(289.5, 88)	14.7
3	(250, 110)	(245.7, 112.5)	11.6	(200, 160)	(192.4, 158.9)	8.5
4	(200, 100)	(192.5, 101.6)	9.3	(200, 60)	(190.6, 57.9)	11.8
5	(300, 90)	(288.3, 88.7)	13.5	(250, 120)	(241.8, 119)	9.3
